# Machine learning prediction of dual and dose-response effects of flavone carbon and oxygen glycosides on acrylamide formation

**DOI:** 10.3389/fnut.2022.1042590

**Published:** 2022-11-30

**Authors:** Laizhao Wang, Fan Zhang, Jun Wang, Qiao Wang, Xinyu Chen, Jun Cheng, Yu Zhang

**Affiliations:** ^1^Zhejiang Key Laboratory for Agro-Food Processing, National-Local Joint Engineering Laboratory of Intelligent Food Technology and Equipment, Integrated Research Base of Southern Fruit and Vegetable Preservation Technology, Zhejiang International Scientific and Technological Cooperation Base of Health Food Manufacturing and Quality Control, College of Biosystems Engineering and Food Science, Zhejiang University, Hangzhou, China; ^2^Fuli Institute of Food Science, Zhejiang University, Hangzhou, China; ^3^Ningbo Research Institute, Zhejiang University, Ningbo, China

**Keywords:** acrylamide formation, flavone carbon and oxygen glycosides, dual effect, flavonoid structure, least squares support vector regression

## Abstract

**Introduction:**

The extensive occurrence of acrylamide in heat processing foods has continuously raised a potential health risk for the public in the recent 20 years. Machine learning emerging as a robust computational tool has been highlighted for predicting the generation and control of processing contaminants.

**Methods:**

We used the least squares support vector regression (LS-SVR) as a machine learning approach to investigate the effects of flavone carbon and oxygen glycosides on acrylamide formation under a low moisture condition. Acrylamide was prepared through oven heating *via* a potato-based model with equimolar doses of asparagine and reducing sugars.

**Results:**

Both inhibition and promotion effects were observed when the addition levels of flavonoids ranged 1–10,000 μmol/L. The formation of acrylamide could be effectively mitigated (37.6%–55.7%) when each kind of flavone carbon or oxygen glycoside (100 μmol/L) was added. The correlations between acrylamide content and trolox-equivalent antioxidant capacity (TEAC) within inhibitory range (*R*^2^ = 0.85) had an advantage over that within promotion range (*R*^2^ = 0.87) through multiple linear regression.

**Discussion:**

Taking ΔTEAC as a variable, a LS-SVR model was optimized as a predictive tool to estimate acrylamide content (*R*^2^_inhibition_ = 0.87 and *R*^2^_promotion_ = 0.91), which is pertinent for predicting the formation and elimination of acrylamide in the presence of exogenous antioxidants including flavonoids.

## Graphical Abstract

Experimental procedure chart. 
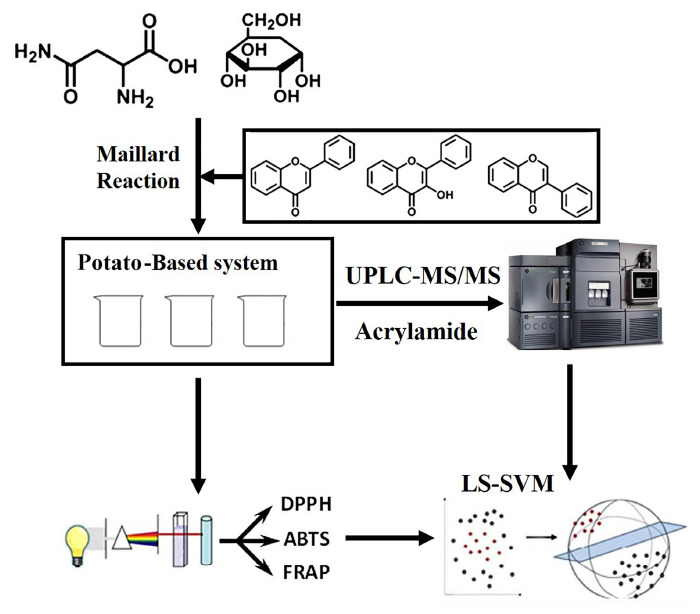

## Introduction

Acrylamide, a recognized food endogenous contaminant, was first discovered in baked and fried foods *via* the Swedish National Food Administration (SNFA). International Agency for Research on Cancer has classified acrylamide as a probable carcinogen to humans (Group 2A substance) (1). Numerous laboratories and institutes have been devoted to investigating the formation and reduction of acrylamide for its widely acknowledged neural, genetic and reproductive toxicities (2, 3). Maillard reaction between the amino acid asparagine and reducing sugars can generate acrylamide within a wide range, depending on reaction time, reaction temperature, pH, etc. (4, 5). Several key intermediates like Schiff base, Amadori products and 3-aminopropionamide have been verified to contribute to the generation of acrylamide (6).

Maillard reaction, the way to the formation of acrylamide, usually occurs during heat processing such as frying, baking and roasting, which is actually under a low-moisture condition. Water activity is an important index in food matrices due to its affection of lipid oxidation rates, the growth rate of food microorganism, browning rate and enzyme activity. The Maillard reaction usually occurs at a water activity of 0.3–0.7 with the maximum reaction rate also being affected by the type of food (7), which indicates water activity can influence acrylamide formation. Some previous studies have revealed that low initial water activity of the system results in a high formation of acrylamide, pointing out that acrylamide formation occurs to a large extent only when the moisture content is below 5% (8, 9).

Given that acrylamide is a Maillard reaction associated product, the major challenge is to simultaneously reduce the formation of acrylamide and remain original color, taste, texture and flavor properties in food (3). Three major mitigation recipes were recommended to reduce acrylamide formation, including the alteration of raw materials, the optimization of heat processing parameters, and the addition of exogenous substances (10). Several studies have found that herbal extracts could effectively inhibit the generation of acrylamide (11). For instance, the formation of acrylamide can be reduced by green tea extracts in fried potatoes (12) and fried chicken drumsticks and chicken wings (13). Flavone carbon and oxygen glycosides inhibit the acrylamide generation by trapping carbohydrates and decreasing lipid oxidation presumably (14). Hitherto, few studies have discussed the effect of flavone carbon and oxygen glycosides on the acrylamide reduction in a low-moisture system.

Many studies have found different effects of various herbal extracts on acrylamide formation in the low-moisture system. Our previous study reported the extracts from both bamboo leaves and green tea could inhibit the acrylamide generation (15). However, oleuropein as a typical polyphenolic compound could promote the generation of acrylamide although many phenolic acids such as caffeic acid, chlorogenic acid, ellagic acid, epicatechin acid, punicalagin and tyrosol could reduce the formation of acrylamide in an asparagine-fructose model system (16). These results indicated that natural antioxidants may possess both reduction and promotion effects on the acrylamide formation. However, it is still unclear how typical natural antioxidants exert their dose-dependent way to the effect on the formation of acrylamide. Additionally, previous studies showed the valuable application of artificial intelligence in food research field (17, 18). Support vector regression (SVR) has unique advantages that it does not need a large number of training samples for developing models and is not affected by the presence of outliers (19).

In the current study, we aim to investigate and compare the dual effect of two typical kinds of flavonoids, flavone carbon and oxygen glycosides, as the representative of natural antioxidants on the acrylamide generation, and employ the machine learning models to associate the concentration of acrylamide with the antioxidant properties of Maillard reaction products (MRP) and predict the chemoprevention of acrylamide.

## Materials and methods

### Chemicals and materials

Apigenin-7-O-glucoside, luteolin-7-O-glucoside, and luteolin-4′-O-glucoside were supplied by Extrasynthese Co. (Lyon, France). Homoorientin, orientin, isovitexin, and vitexin were isolated and purified from antioxidants of bamboo leaves by preparative high-performance liquid chromatography (HPLC) on the basis of our previous studies (20). Acrylamide, L-asparagine monohydrate, D-(+)-glucose monohydrate, 2,2-diphenyl-1-picrylhydrazyl (DPPH), 2,2-azino-bis-(3-ethylbenzothiazoline-6-sulfonic acid) (ABTS), 6-hydroxy-2,5,7,8-tetramethylchromane-2-carboxylic acid (trolox), potassium persulfate and 2,4,6-tri(2-pyridyl)-s-triazine (TPTZ) were purchased from Sigma-Aldrich (St. Louis, MO, USA). D_3_-labeled acrylamide (isotopic purity ≥ 99%) was supplied by Cambridge Isotope Laboratories (Andover, MA, USA). Atlantis potato powder was purchased from Sanjiang (Group) Potato Products Co., Ltd. (Lintao, Gansu, China).

### Preparation of low-moisture Maillard reaction system

A potato-based equimolar asparagine-reducing sugar system was used to investigate the role of flavone carbon and oxygen glycosides. Oven heating, a high-speed and short-time internal heating method, was used to reach the heating temperature that the Maillard reaction needed according to previous work (20). Potato powder (50 g) and the reactant powder of asparagine (0.14 mol) and glucose (0.14 mol) were grinded carefully to ensure adequate reaction. On the basis of our previous study using HPLC (21), the original concentrations of asparagine (0.037 mg) and glucose (0.015 mg) measured in potato powder (100 mg) could be negligible compared with the equimolar addition concentrations of asparagine (1 mmol) and glucose (1 mmol) used in the experiment.

### Effects of flavone carbon and oxygen glycosides on the formation of acrylamide

On account of mimicking the reduction of acrylamide by flavonoids in the asparagine-glucose reaction system, gradient addition levels of each kind of flavone carbon and oxygen glycosides were added into the treatment groups, while phosphate buffer (0.1 mol/L, pH 6.80) was added into the control groups. A series of working solutions of standards (100, 500, 1,000, 5,000, 10,000, 50,000, 100,000, 500,000, and 1,000,000 μmol/L) was diluted in phosphate buffer. Then, 100 μL working solution was added into the low-moisture system. The oven was adjusted to the predesigned temperature (180°C). The mixed reactant powders were hermetically sealed in hard beakers and then went through the oven-heating process under the selected heating temperature (180°C) at a set time (15 min), the optimal heating conditions when the acrylamide generation reached the maximal level were used according to previous study (22). After the heating stage, the hard-beakers were carefully taken out from the oven and transferred into an ice bath immediately to stop further reaction. The cooled reaction products were stored at 4°C to avoid Maillard reaction for further use. The concentrations of flavone carbon and oxygen glycosides in reaction solutions (10 ml) for the sample treatment were 1, 5, 10, 50, 100, 500, 1,000, 5,000, and 10,000 μmol/L in different treatment groups.

### Quantification of acrylamide concentration in Maillard reaction products by ultra-high performance liquid chromatography tandem mass spectrometry

The method used for the sample treatment and the analysis of acrylamide by UHPLC-MS/MS were conducted according to our previous study (23). Firstly, PBS (10 ml, 0.1 mol/L, pH 6.8) was used to dissolve the cooled MRP, followed by the addition of D_3_-acrylamide (500 μL, 2 μg/ml). Then, the mixed solution (2 ml) was liquid-liquid extracted using ethyl acetate (2 ml) thrice. The organic phase was fused and dried with nitrogen gas. Then, 1.5 ml water was used to redissolve the residue and Oasis HLB cartridges (6 ml, 200 mg; Waters Technology, Milford, MA, USA) were used for solid phase extraction (SPE) purification according to previous study (22). Finally, the collected eluent was used for injection for the UHPLC-MS/MS analysis.

We used an Acquity ultra-high performance liquid chromatograph equipped with vacuum degasser, sample tray and autosampler (Waters, Milford, MA, USA) to conduct the UHPLC analysis. We completed the UHPLC separation by using a Waters UPLC BEH C_18_ column (50 mm × 2.1 mm i.d., 1.7 μm). Tandem mass spectrometry was performed on a Quattro Ultima Pt tandem mass spectrometer (Micromass Company Inc., Manchester, UK) using an electrospray source in a positive ion mode (ESI+) to detect characteristic precursors and product ions of acrylamide. The conditions used for the electrospray source were as follows: capillary voltage, 3.5 kV; cone voltage, 50 V; source temperature, 100°C; desolvation gas temperature, 350°C; desolvation gas flow, 400 L/h nitrogen; cone gas flow, 45 L/h nitrogen; argon collision gas pressure to 3 × 10^–3^ mbar for MS/MS, which gave a highest acrylamide response in this study. The collision energy (CE) was optimized for each multiple reaction monitored (MRM) transition (20).

### Evaluation of antioxidant properties of Maillard reaction products

Three antioxidant evaluation methods [DPPH, ABTS, and ferric ion reducing antioxidant power (FRAP) assays] were conducted to investigate the antioxidant characteristics of MRP. (i) DPPH assay was used to determine the radical scavenging activity of MRP by a spectrophotometer (Shengke Instrument Equipment Co., Ltd., Shanghai, China) based on a modified procedure (24). An aliquot of sample (20 μL) after SPE treatment was reacted with 5 ml of DPPH solution (74 mg/L) at room temperature in the dark for 1 h. UV-Vis readings were recorded at 520 nm. A series of aqueous trolox solutions (0–0.4 mmol/L) were measured in the same condition and used to calibrate the results. (ii) ABTS assays was conducted according to a previous work (25). An aliquot of sample treatment solution (5 μL) was added into 5 ml of ABTS+ solution (70 μmol/L) and reacted for 20 min. The decline of absorbance was recorded at 730 nm in 10 min. Aqueous trolox solutions (0–0.12 mmol/L) were measured and used to calibrate the results. (iii) FRAP assay was also conducted on the basis of previous study with modifications (21). The FRAP reagent included TPTZ (10 mmol/L), FeCl_3_ (20 mmol/L), and acetate buffer (0.3 mol/L) in a certain ratio (1:1:10). SPE-treated sample (6 μL) was added into 4.5 ml FRAP working solution. Then, the absorbance was measured at 595 nm after vortex mixing. Calibration procedure was conducted using a linear range of 0–0.3 mmol/L Trolox. These results were all expressed as Trolox equivalent antioxidant capacity (TEAC) and figured as μmol trolox per ml.

### Least squares support vector regression modeling and statistical analysis

All the experiments were performed in triplicate. The concentrations of acrylamide were expressed as the mean ± standard deviation (SD) values. This study measured the inhibitory effects of four flavone carbon glycosides and three flavone oxygen glycosides at 10 different addition levels for each on the formation of acrylamide in MRP. Given that each experiment was performed in triplicate, we thus obtained 210 groups of experimental data. Data from each group contained the concentration of acrylamide generated from the low-moisture system and three TEAC values from three different antioxidant evaluation methods.

The LS-SVR modeling approach was used for predicting the effect of flavone carbon and oxygen glycosides on acrylamide generation *via* triple TEAC variables. The training and predicting data sets had been first assigned. Data were divided into two parts according to the effect on acrylamide generation. In each part, we used the software of Matlab R2010b (The MathWorks, Natick, MA, USA) to sort data. The middle one of every three data was taken out as the prediction of the effect, and the rest were applied for building the model. The LS-SVR model was operated *via* the Matlab R2010b combining with LS-SVR toolbox (LS-SVR v 1.5, Suykens, Leuven, Belgium).

To compare the regression efficiency of LS-SVR approach with the routine model, data were simultaneously fitted using the multiple linear regression (MLR) and partial least squares regression (PLS) models, based both on linear model and quadratic model. Model performance was evaluated using determination coefficient of calibration (*R*^2^C), determination coefficient of validation (*R*^2^V), determination coefficient of prediction (*R*^2^P), root mean square error of calibration (RM_SEC_), root mean square error of validation (RM_*SEV*_), and root mean square error of prediction (RM_SEP_) as indicators. Statistical significance of differences was evaluated by two-way ANOVA test and correlations were evaluated by calculating the Pearson’s correlation coefficient using SPSS (version 22.0, IBM Corp., Armonk, NY, USA). Differences were considered significant at *p* < 0.05 in all statistical tests.

## Results and discussion

### Comparison of the dose-response effects of flavone carbon and oxygen glycosides on acrylamide formation

**FIGURE 1:**
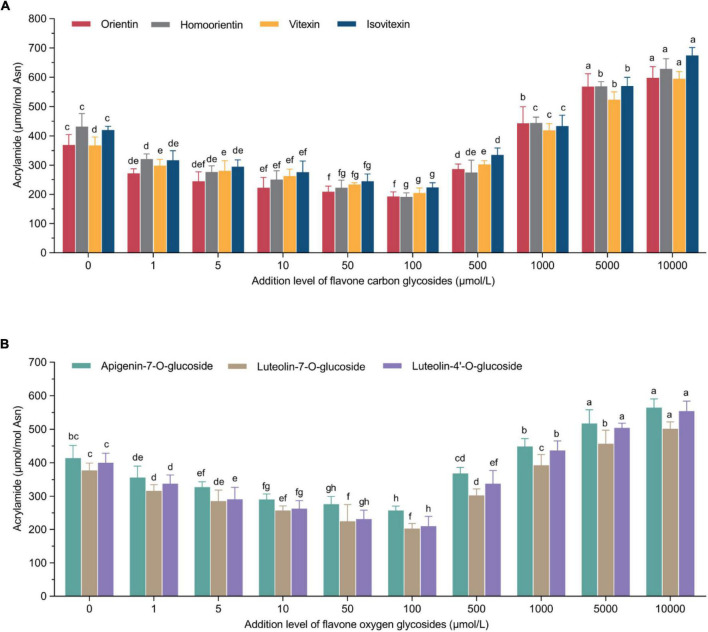
U-shaped correlations between acrylamide concentrations and treatment levels of **(A)** flavone carbon glycosides or **(B)** flavone oxygen glycosides. Data are shown as the mean ± SD (*n* = 3). Values with different letters indicate significant differences between different additional levels at the same flavonoid (*P* < 0.05).

To observe the relationships between the formation of acrylamide and the addition of flavone carbon and oxygen glycosides in the low-moisture system, a dose-response study was conducted in the potato-based system. Both inhibition and promotion effects were observed when the addition levels of flavonoids ranged from 1 to 10,000 μmol/L (Figure 1). In addition, there was an effect of types and addition levels of flavone carbon and oxygen glycosides on the production of acrylamide, which is shown in Supplementary Table 1. In detail, a significant dose-response inhibitory effect could be found by the addition of flavonoids within a range of 1–100 μmol/L. However, the inhibitory tendency of acrylamide formation decreased when the addition level surpassed 100 μmol/L. What’s more, the acrylamide content was dramatically promoted when the addition levels of flavonoids ranged 1,000–10,000 μmol/L. As a result, the optimal addition level of flavone carbon and oxygen glycosides with their ability of reducing the acrylamide content was 100 μmol/L, at which orientin, homoorientin, vitexin and isovitexin among flavone carbon glycosides exerted their maximal inhibitory rates of 47.9%, 55.7%, 44.3%, and 46.9%, respectively, while in the mean time apigenin-7-O-glucoside, luteolin-7-O-glucoside and luteolin-4′-O-glucoside among flavone oxygen glycosides maximally reduced the acrylamide content by 37.6%, 46.2%, and 47.5%, respectively. Furthermore, the maximal promotion effects of orientin, homoorientin, vitexin, isovitexin, apigenin-7-O-glucoside, luteolin-7-O-glucoside and luteolin-4′-O-glucoside could reach 1.62-fold, 1.45-fold, 1.62-fold, 1.61-fold, 1.36-fold, 1.32-fold, and 1.38-fold for the acrylamide concentration when compared to the control group at their addition levels of 10,000 μmol/L. These unexpected adverse effects seemed like violating the definition of flavone carbon and oxygen glycosides as acrylamide inhibitors. Diverse factors are put forward to clarify the so-called “antioxidant paradox” contributing to the dual effect of flavonoids on the formation of acrylamide, including the intrinsic properties of antioxidants, the antioxidants of products and the antioxidant activity of model system. Some promotion mechanisms have been proved to take part in the reaction, including trapping carbonyl compounds (26) and preventing against lipid oxidation (27). However, flavonoids can simultaneously provide carbonyl groups for acrylamide formation (28), inhibit the acrylamide elimination (29) and accelerate the conversion from 3-APA to acrylamide (30). Previous study similarly reported the phenomenon of both promotion and reduction of acrylamide content in wheat bread by the addition of rosemary, dittany and catechin compounds in a dose-dependent way, and clarified the role of rosemary extract and catechin compounds in the acrylamide formation during the Maillard reaction (31). The addition of polyphenols (0.1%, m/m) including catechin, quercetin, gallic acid, ferulic acid and caffeic acid significantly reduced acrylamide by 16.2–92.5% in a model wheat bread system, but in the case of quercetin, a promotion effect was observed with the increase of its concentration (32). Yuan et al. (33) reported that not only the type but also the concentration of phenolic compounds might play a significant role in the formation of acrylamide. Especially, ascorbic acid, a typical water-soluble antioxidant, exerted an inhibitory effect up to 58% within an addition range of 0.2–1.2%, whereas showed a promotion effect with the addition percentage of 1.5% in an asparagine-glucose model system. Furthermore, an equimolar asparagine-glucose model system indicates that different vitamin A and E homologue concentrations could determine their functionality either as antioxidants or pro-oxidants (34).

For the effect of flavonoids, we initially revealed that a flavonoid-rich antioxidant extract from bamboo leaves effectively reduced the formation of acrylamide in cookies in a non-linear manner (35). Among different types of flavonoids, we also demonstrated that catechin compounds as flavanols and their derivatives showed better but non-linear inhibitory effect on the reduction of acrylamide in a microwave heating model system (36). Qi et al. (37) investigated the effect of proanthocyanidins with different oligomer structures in a fried potato crisp model and observed that oligomer-1, oligomer-2 and oligomer-4 proanthocyanidins (100 μg/ml) could effectively reduce the formation of acrylamide by 41.2%, 46.1%, and 39.6%, respectively, but they did not observe any promotion effect. In view of chemical structures, B-type proanthocyanidins had stronger inhibitory effect than A-type, but unit composition and degree of polymerization had no significant inhibitory effect based on the same mass. In the current study, all the selected flavonoids showed a maximal inhibitory effect when the addition level was 100 μmol/L. In detail, the inhibitory effect of four flavone carbon glycosides ranged 44.3–55.7%, in which homoorientin exerted a maximal effect (*n* = 3, Figure 1A); the inhibitory effect of three flavone oxygen glycosides spanned 37.6–47.5%, in which the mitigation effect of luteolin-4′-O-glucoside should be preferred (*n* = 3, Figure 1B). The above results recommended an overall better inhibitory effect of flavone carbon glycosides on the acrylamide generation than the effect of flavone oxygen glycosides, indicating a role of phenolic hydroxyl groups in reducing the acrylamide formation rather than alcoholic hydroxyl groups in the chemical structures of flavone glycosides. As a matter of fact, various activities of flavonoids and polyphenols hinges on their functional groups in the chemical structures such as number and position of phenolic hydroxyl groups, steric effects, and molecular properties (38). Santos et al. (39) investigated the association between chemical structures and antioxidant capacity of hydrolysable tannins and flavonoids by DPPH and ORAC assays and molecular descriptors obtained through density functional theory, and found that the cluster with more active compounds was mostly comprised of monomeric and dimeric ellagitannins. What’s more, the effect of heating processing plays a significant role in the antioxidant activities of flavonoids. Depending on Arrhenius law, the activation energy was 51.4 kJ/mol for luteolin and 120 kJ/mol for luteolin-7-O-glucoside, indicating glycosylated flavonoids may be more resistant than aglycon flavonoids to heat treatment (40). In the current study, although flavone carbon glycosides and oxygen glycosides have the same aglycone structure, the former performed better in the inhibition of acrylamide formation than the latter (e.g., isovitexin > apigenin-7-O-glucoside), which may be ascribed to the difference of phenolic hydroxyl group numbers. In other words, the insertion of oxygen glucoside eliminates one phenolic hydroxyl of the aglycon, while the introduction of carbon glucoside maintains the original number of phenolic hydroxyls compared with the aglycon.

### Antioxidant properties of Maillard reaction products and its association with inhibition and promotion effects

**FIGURE 2:**
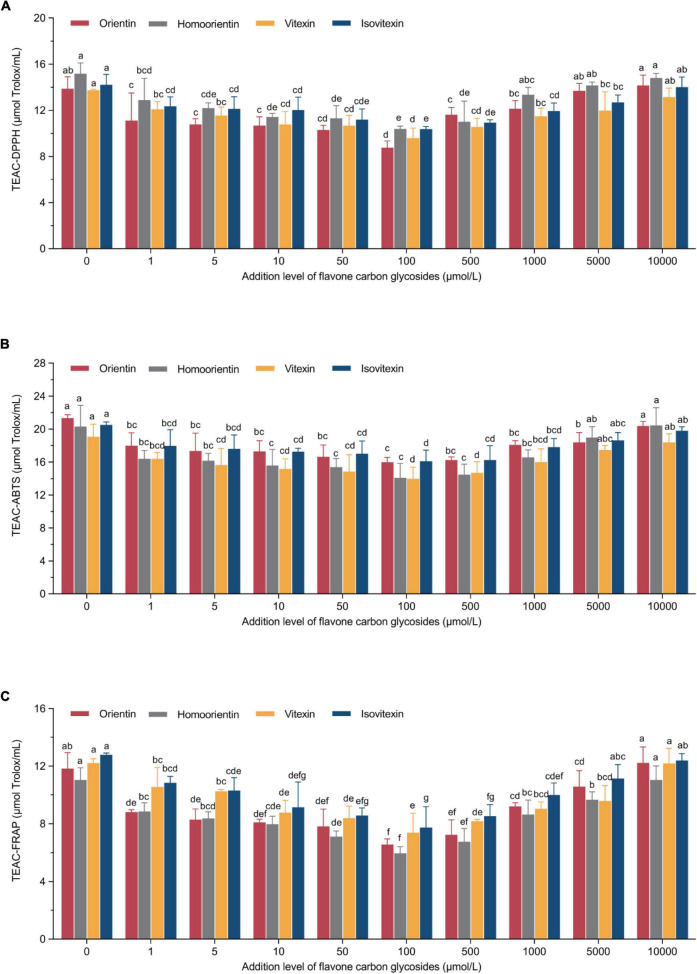
U-shaped correlations between treatment levels of flavone carbon glycosides and **(A)** TEAC-DPPH values, **(B)** TEAC-ABTS values, or **(C)** TEAC-ferric ion reducing antioxidant power (FRAP) values in Maillard reaction products. Data are shown as the mean ± SD (*n* = 3). Values with different letters indicate significant differences between different additional levels at the same flavonoid (*P* < 0.05).

**FIGURE 3:**
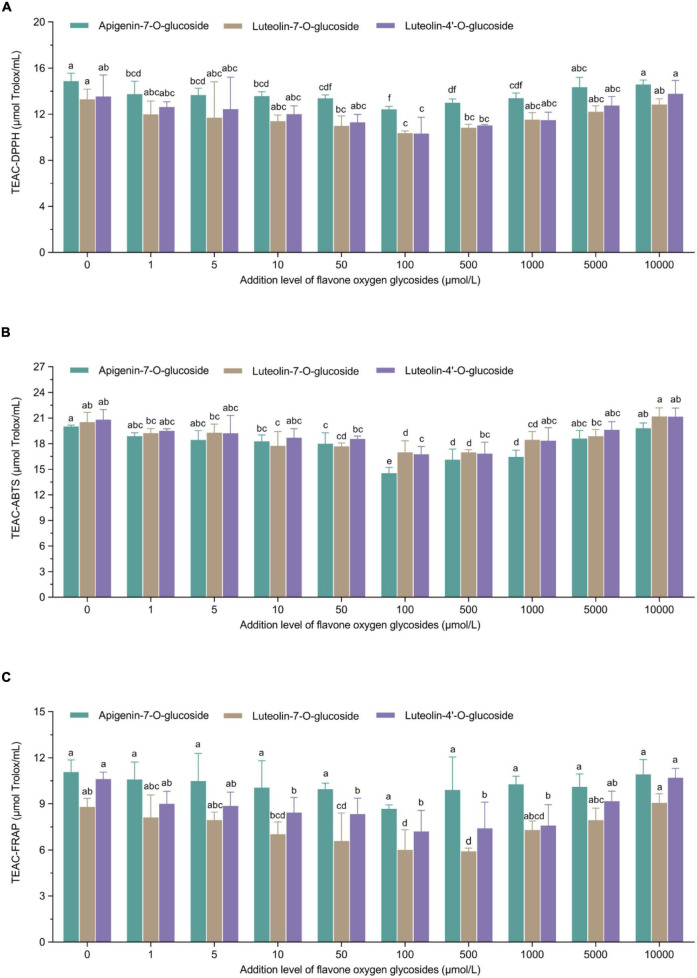
U-shaped correlations between treatment levels of flavone oxygen glycosides and **(A)** TEAC-DPPH values, **(B)** TEAC-ABTS values, or **(C)** TEAC-FRAP values in Maillard reaction products. Data are shown as the mean ± SD (*n* = 3). Values with different letters indicate significant differences between different additional levels at the same flavonoid (*P* < 0.05).

The principle of these representative assays is to measure the antioxidant properties *via* the single electron transfer-induced elimination of radicals (41), and the mechanism can be used to determine the antioxidant role of flavone carbon and oxygen glycosides used in the current study. The parabolic-shape correlations between TEAC_DPPH_/TEAC_ABTS_/TEAC_FRAP_ values and the addition levels of flavone carbon and oxygen glycosides is shown in Figures 2, 3. We found the lowest TEAC values when the addition levels of all the flavone carbon and oxygen glycosides were 100 μmol/L, which was used to achieve the maximal inhibition effect on acrylamide formation. In addition, we also identified a significant correlation between acrylamide contents and TEAC values in MRP using Pearson’s correlation test (*R*_TEAC–DPPH_ = 0.68–0.89, *R*_TEAC–ABTS_ = 0.48–0.88, and *R*_TEAC–FRAP_ = 0.58–0.84), which is presented as heat maps in Supplementary material (Supplementary Figure 1). These results were in agreement with some previous studies, reporting similar associations between the acrylamide concentrations and the total antioxidant capacity in model reaction system and real food (21, 36, 42). These results demonstrated that the TEAC values of MRP might be an appropriate predictor for the acrylamide generation and its inhibition/promotion change by the addition of flavone carbon and oxygen glycosides.

Our study linked the addition of flavone glycosides with the TEAC values of MRP. Based on the possible formation of antioxidant-sugar adducts in model systems, phenolic antioxidants such as flavonoids may interact with sugar fragments and/or reactive carbonyl compounds, generate various adducts through electrophilic aromatic substitution reactions and thus reduce the formation of acrylamide in model Maillard reaction systems (43). However, several studies reported positive, negative or no effects and thus revealed ambiguous role of phenolic and other antioxidants on the formation of acrylamide when investigating their effect on acrylamide (42, 44, 45). Therefore, the variability in experimental parameters such as thermal conditions, food matrix and the addition level of antioxidants may explain the conflicting observations regarding their efficacy as acrylamide inhibitors and/or promotors (43). For instance, the co-formation profile of both toxic acrylamide and beneficial antioxidants changed during different stages of coffee roasting process, indicating the effect of thermal processing and food matrix on the correlation between acrylamide formation and antioxidant property of MRP (46). Although numerous studies reported dose-dependent reduction or mitigation effect of various phenolic antioxidants on the formation of acrylamide in both model system and food (32, 47), we uniquely observed non-linear U-shape tendency of both antioxidant capacity of MRP and acrylamide concentrations with the increase of addition levels of all the selected flavone glycosides. Thermal processing with the addition of flavone glycosides exhibits higher antioxidant capacity due to the generation of MRP such as melanoidins but simultaneously produces related harmful compounds such as acrylamide, indicating we should get insight into the optimal heat processing procedures based on simultaneous consideration of bioactive compounds, sensory characteristics, and safety (48). Meanwhile, compared with the control group, the addition of flavone glycosides at their high levels may promote the formation of acrylamide but not enhance the antioxidant capacity of MRP, suggesting these types of flavone glycosides may reduce the generation of melanoidins, which can be used as a color indicator for controlling the thermal processing and Maillard reaction (49). Further studies are still warranted to unravel how flavone carbon and oxygen glycosides control both acrylamide formation and antioxidant capacity of MRP.

### Prediction of reduction/promotion effect of flavone carbon and oxygen glycosides on acrylamide formation based on least squares support vector regression modeling

**FIGURE 4:**
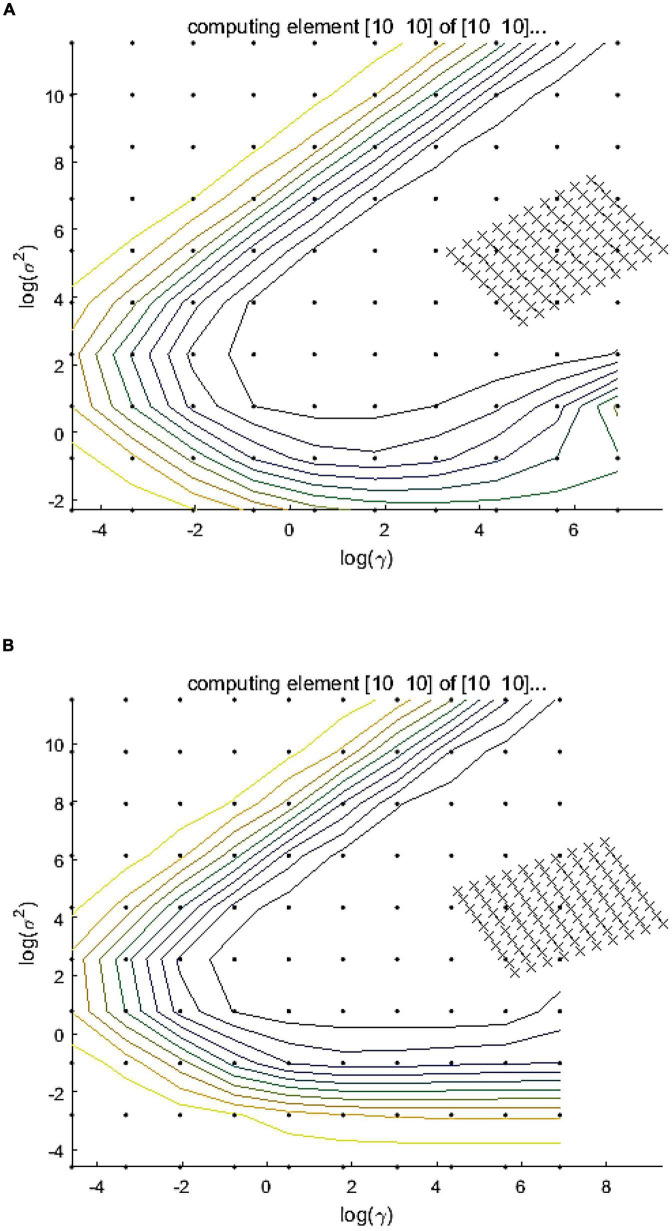
The LS-SVR models were established to predict the reduction effect of acrylamide, and the model performance including the optimization of γ and δ^2^ was investigated when the addition level of flavonoids was **(A)** in the range of 1–100 μmol/L (inhibition range) or **(B)** in the range of 100–10,000 μmol/L (promotion range).

In consideration of the correlation between the inhibition/promotion effect of flavonoids on the acrylamide formation and the antioxidant capacity of MRP, the least squares support vector regression (LS-SVR) models were used to predict the inhibition/promotion effect using triple antioxidant measurement variables. By comparison against LS-SVR and PLS models, consensus LS-SVR model gives more stable and accurate prediction results. Therefore, consensus strategy like LS-SVR modeling may be a promising way for quantitative modeling of complex samples (50). The antioxidant properties of MRP were regarded as three independent variables and the corresponding acrylamide concentration was served as dependent variable. We used the radial basis function as a kernel function in the current study. γ and δ^2^, two important parameters, were determined before applying LS-SVR model. Among these, γ, called penalty factor, minimizes the structural and empirical risk and also plays a leading part in the improvement of the generalization in this model. δ^2^, called the width of the kernel function, controls the regression error and reflects the sensitivity given by the input variables. In this study, the parameters were selected by using grid searching technique to ensure the accuracy of the model. According to previous experiments, the ranges of γ and δ^2^ for acrylamide concentration are set and shown in Table 1 and Supplementary Table 2. The optimization procedures are shown in Figure 4 and Supplementary Figure 2. As shown in Table 1, the *R*_c_^2^ and *R*_p_^2^ of the inhibition range of acrylamide were 0.87 and 0.82, respectively, while they were 0.91 and 0.77 when the prediction fell into the promotion range of acrylamide, respectively.

**TABLE 1:** Results of LS-SVR models for predicting acrylamide concentrations based on antioxidant properties.

Component	Range of γ	Optimal γ	Range of δ^2^	Optimal δ^2^	*R*_c_^2^	SEC (%)	*R*_*p*_^2^	SEP (%)	RPD
Acrylamide (inhibition range)	0.01–100,000	1554.9	0.01–100,000	447.0	0.87	24.41	0.82	29.04	2.35
Acrylamide (promotion range)	0.01–100,000	490.1	0.01–100,000	60.4	0.91	42.80	0.77	67.94	2.10

**FIGURE 5:**
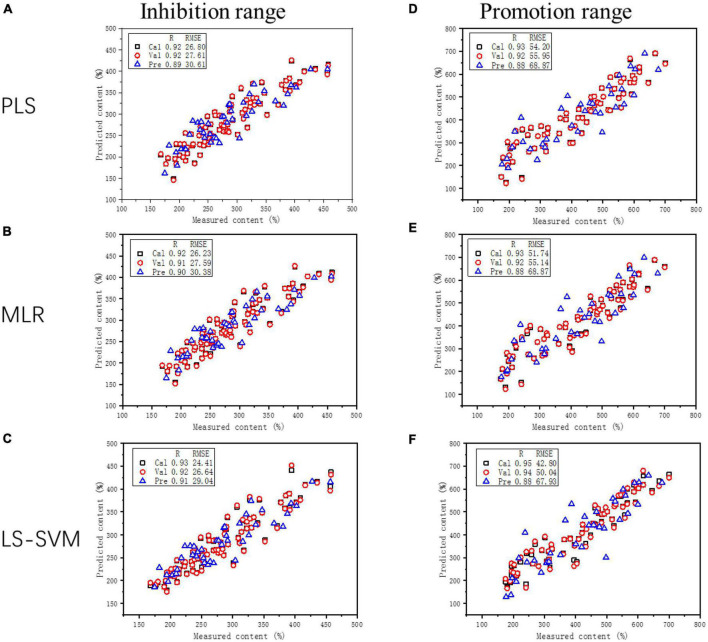
Graphs of three models (PLS, MLR, and LS-SVR) for estimating the correlation between predicted concentrations and measured values and showing both inhibition and promotion effects on acrylamide formation. Inhibition range of PLS, MLR, and LS-SVR model are presented in panels **(A–C)**, respectively; Promotion range of PLS, MLR, and LS-SVR model are presented in panels **(D–F)**, respectively.

Furthermore, we compared the LS-SVR model with other models such as PLS and MLR for predicting both inhibition and promotion effects of flavone glycosides on the generation of acrylamide. The graphs of three linear models are shown in Figure 5 and the relevant data about inhibition or promotion rates are shown in Table 2. The graphs of three quadratic models are shown in Supplementary Figure 3 and the relevant data are shown in Supplementary Table 3. When *R*^2^C and *R*^2^P values are larger and the difference between them is smaller, and RM_SEC_ and RM_SEP_ values are smaller and the difference between them is smaller, then the performance of the corresponding model is better. In the prediction of inhibition range, LS-SVR model (0.87 of *R*^2^C, 0.82 of *R*^2^P, 24.41 of RM_SEC_ and 29.04 of RM_SEP_) exhibited better predictive ability compared with PLS model (0.84 of *R*^2^C, 0.79 of *R*^2^P, 26.8 of RM_SEC_ and 30.61 of RM_SEP_) and MLR model (0.85 of *R*^2^C, 0.80 of *R*^2^P, 26.23 of RM_SEC_ and 30.38 of RM_SEP_). The similar phenomenon occurred in respect of the prediction of promotion range and dual effect range. It is clear that the quadratic model predictions seem unqualified. In conclusion, compared with the traditional multiple linear regression model including PLS and MLR, LS-SVR model can better predict inhibition and promotion effects of flavone carbon and oxygen glycosides on the formation of acrylamide.

**TABLE 2:** Statistical variables of training and testing data of inhibition or promotion rate (%) in the PLS, MLR, LS-SVR models.

	Model	*R*^2^C	RM_SEC_	*R*^2^V	RM_SEV_	*R*^2^P	RM_EP_
Inhibition range	PLS	0.84	26.80	0.84	27.61	0.79	30.61
	MLR	0.85	26.23	0.84	27.59	0.80	30.38
	LS-SVR	0.87	24.41	0.85	26.64	0.82	29.04
Promotion range	PLS	0.86	54.20	0.85	55.95	0.78	67.55
	MLR	0.87	51.74	0.85	55.13	0.77	68.87
	LS-SVR	0.91	42.80	0.88	50.04	0.77	67.94

## Conclusion

In this work, we found that flavone carbon and oxygen glycosides have both inhibition and promotion effects on the formation of acrylamide using an asparagine-glucose model under a low-moisture system. When the addition levels ranged 1–100 μmol/L, the flavone carbon and oxygen glycosides conducted inhibition for the acrylamide formation which could reach the maximum inhibition rate (37.5–55.7%) till the addition level was 100 μmol/L. On the other hand, when the addition concentration ranged 1,000–10,000 μmol/L, the system demonstrated promotion effect and the maximal promotion effect could reach 1.33-fold to 1.62-fold for control while the addition level is 10,000 μmol/L. Taking the phenolic hydroxyl group numbers into consideration, the inhibition/promotion effect may be related to the number of phenolic hydroxyl groups and the differences of thermal resistance. The reduction or promotion effects of flavone carbon and oxygen glycosides could be affected by the addition level and the chemical structure. Our study here also identified a linear relationship between the acrylamide content and the antioxidant capacities of MRP. Using TEAC values as variables, a LS-SVR model could serve as a predictive tool for estimating the generation and reduction of acrylamide in a low-moisture system. Compared with the traditional MLR models, the machine learning approaches like LS-SVR could fit a better correlation coefficient to predict the change of acrylamide content in the food system. However, the mechanistic role of flavone carbon and oxygen glycosides in affecting acrylamide formation still remains unclear. Further studies should continuously focus on the inhibition or promotion effect of flavone carbon and oxygen glycosides on the kinetics of acrylamide. Meanwhile, further optimized prediction by various machine learning approaches may emphasize the interaction between confounding factors and the estimated results, and focus on the fusion of machine learning and experimental design to precisely estimate the reduction/promotion of typical food contaminants by accelerating and improving data processing and real-time decision-making.

## Data availability statement

The raw data supporting the conclusions of this article will be made available by the authors, without undue reservation.

## Author contributions

YZ conceived and designed the study, was the guarantor of the work and, as such, has full access to all the data in the study, takes responsibility for the integrity of the data, and accuracy of the data analysis. LW, FZ, JW, QW, XC, and JC performed the experiment. QW, XC, and JC contributed to data collection. LW and JW drafted the manuscript. QW and XC interpreted the data. LW, FZ, JW, and YZ critically revised the manuscript. All authors read and approved the manuscript.

## Abbreviations

ABTS: 2,2-azino-bis-(3-ethylbenzothiazoline-6-sulfonic acid)
DPPH: 2,2-diphenyl-1-picrylhydrazyl
HPLC: high-performance liquid chromatography
LS-SVR: least squares support vector regression
MRM: multiple reaction monitoring
MRP: Maillard reaction products
SNFA: Swedish National Food Administration
TEAC: trolox equivalent antioxidant capacity
TPTZ: 2,4,6-tri(2-pyridyl)-s-triazine
trolox: 6-hydroxy-2,5,7,8-tetramethylchromane-2-carboxylic acid
UHPLC-MS/MS: ultra-high performance liquid chromatography tandem mass spectrometry
SPE: solid phase extraction.
