# Supplementary material for: Machine learning prediction of dual and dose-response effects of flavone carbon and oxygen glycosides on acrylamide formation
Source: Front Nutr. 2022 Nov 30;9:1042590. doi: 10.3389/fnut.2022.1042590 (PMC9748078; doi:10.3389/fnut.2022.1042590)
Supplement: Supplementary file 1 [file Data_Sheet_1.docx]

**Supplementary Material**

**Machine learning prediction of dual and dose-response effects of flavone carbon and oxygen glycosides on acrylamide formation**

**Laizhao Wang ^1†^, Fan Zhang ^1†^, Jun Wang ^1,2^, Qiao Wang ^1^, Xinyu Chen ^1^, Jun Cheng ^1^, Yu Zhang ^1,2,3^***

^1^ College of Biosystems Engineering and Food Science, National-Local Joint Engineering Laboratory of Intelligent Food Technology and Equipment, Zhejiang Key Laboratory for Agro-Food Processing, Integrated Research Base of Southern Fruit and Vegetable Preservation Technology, Zhejiang International Scientific and Technological Cooperation Base of Health Food Manufacturing and Quality Control, Zhejiang University, Hangzhou, 310058, Zhejiang, China

^2^ Fuli Institute of Food Science, Zhejiang University, Hangzhou, 310058, Zhejiang, China

^3^ Ningbo Research Institute, Zhejiang University, Ningbo, 315100, Zhejiang, China

† These authors contributed equally to this work.

***Correspondence:**

Yu Zhang, e-mail: y_zhang@zju.edu.cn


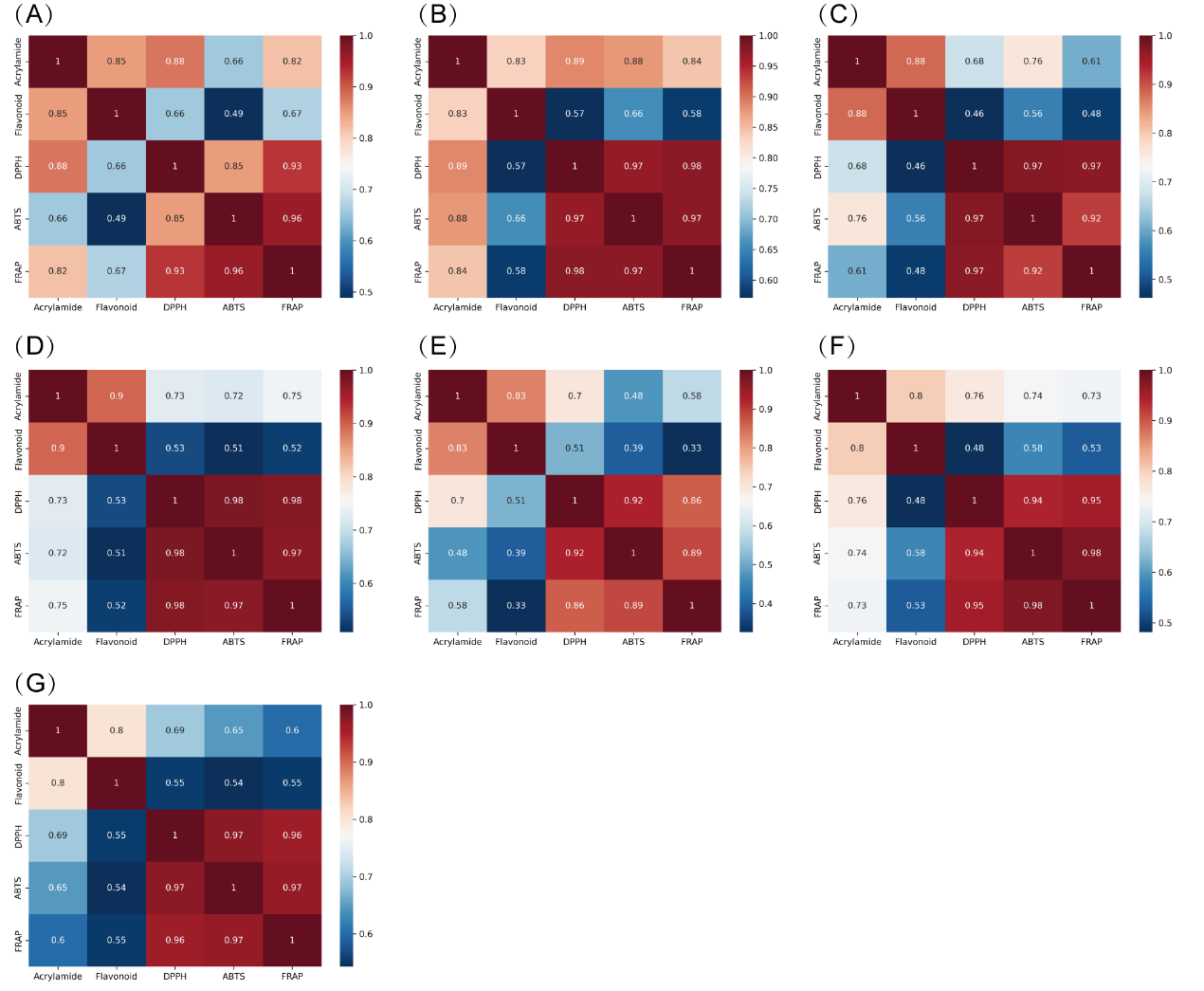


**FIGURE S1**. Correlogram among addition levels of different types of flavonoids, acrylamide concentrations and Trolox equivalent antioxidant capacity values. (A) Orientin, (B) Homoorientin, (C) Vitexin, (D) Isovitexin, (E) Apigenin-7-O-glucoside, (F) Luteolin-7-O-glucoside, or (G) Luteolin-4'-O-glucoside.


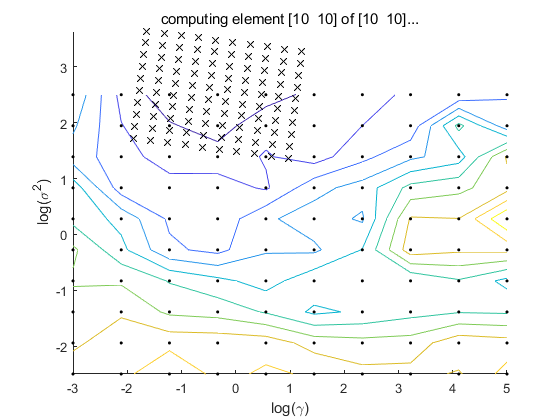


**FIGURE S2.** The LS-SVR models were established to predict the reduction effect of acrylamide, and the model performance including the optimization of γ and δ^2^ was investigated when the addition level of flavonoids was 1-10000 µmol/L (dual effects range).


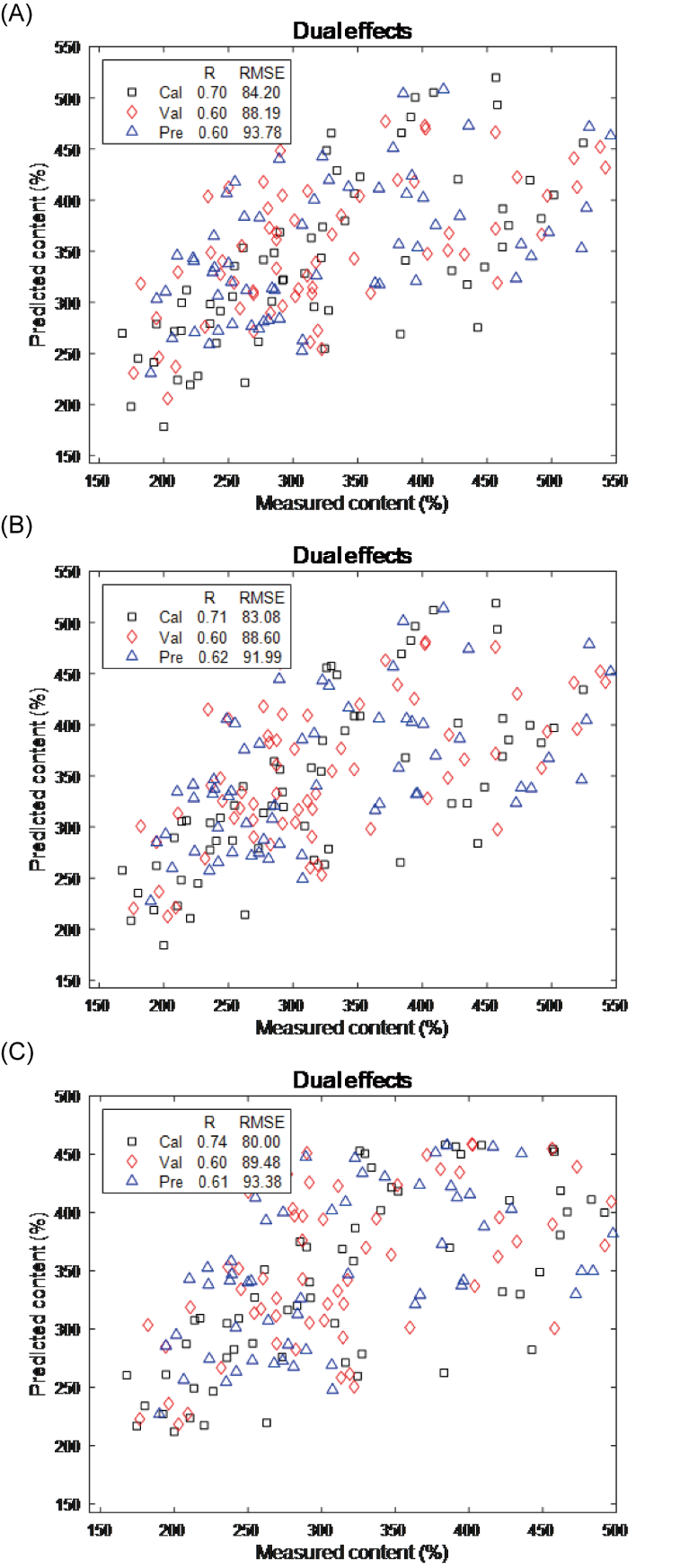


**FIGURE S3.** Graphs of three models (PLS, MLR and LS-SVR) for estimating the correlation between predicted concentrations and measured values and showing dual effects on acrylamide formation. PLS, MLR and LS-SVR model are presented in panels (A), (B) and (C), respectively.

**Table S1**. *P*-values of the two-way ANOVA for the effect of types and addition levels of flavone carbon and oxygen glycosides on acrylamide formation or trolox equivalent antioxidant capacity using DPPH, ABTS or FRAP assay. ^a^NaN, not a number.

|  |  | | Acrylamide | DPPH | ABTS | FRAP |
| --- | --- | --- | --- | --- | --- | --- |
| Flavone carbon glycosides | | Types | 4.30E-04 | 1.54E-04 | 1.48E-09 | 9.29E-09 |
|  |  | Addition levels | 1.38E-23 | 5.17E-12 | 2.09E-15 | 1.75E-16 |
|  |  | Residual^a^ | NaN | NaN | NaN | NaN |
| Flavone oxygen glycosides | | Types | 1.94E-08 | 1.24E-12 | 4.69E-04 | 3.35E-10 |
|  |  | Addition levels | 1.09E-17 | 3.76E-10 | 9.89E-09 | 2.79E-06 |
|  |  | Residual^a^ | NaN | NaN | NaN | NaN |

**Table S2**. Results of quadratic LS-SVR models for predicting acrylamide concentrations based on antioxidant properties

| Range of γ | Optimal γ | Range of δ^2^ | Optimal δ^2^ | *R*_c_^2^ | SEC(%) | *R*_p_^2^ | SEP(%) | RPD |
| --- | --- | --- | --- | --- | --- | --- | --- | --- |
| 0.01-100000 | 0.9 | 0.01-100000 | 16.4 | 0.55 | 80.00 | 0.37 | 93.38 | 1.26 |

**Table S3**. Statistical variables of training and testing data of inhibition or promotion rate (%) from dual effects range in the PLS, MLR, LS-SVR models.

| Model | *R*^2^C | RM_SEC_ | *R*^2^V | RM_SEV_ | *R*^2^P | RM_SEP_ |
| --- | --- | --- | --- | --- | --- | --- |
| PLS | 0.50 | 84.20 | 0.36 | 88.19 | 0.37 | 93.78 |
| MLR | 0.51 | 83.08 | 0.36 | 88.60 | 0.39 | 91.99 |
| LS-SVR | 0.55 | 80.00 | 0.36 | 89.48 | 0.37 | 93.38 |
